# COVID-19 vaccine policy development in a sample of 44 countries – Key findings from a December 2021 survey of National Immunization Technical Advisory Groups (NITAGs)

**DOI:** 10.1016/j.vaccine.2022.11.029

**Published:** 2023-01-16

**Authors:** Anna-Lea Kahn, Christoph A. Steffen, Louise Henaff, Noni E. MacDonald, Christopher Morgan, Ruth Faden, Folake Olayinka, Shalini Desai

**Affiliations:** a Department of Immunization, Vaccines and Biologicals, World Health Organization, Avenue Appia 20, 1211 Geneva, Switzerland; b Dept of Paediatrics, Faculty of Medicine, Dalhousie University, Halifax, NS, Canada; c Jhpiego, The Johns Hopkins University Affiliate, Baltimore, MD, USA; d Nossal Institute, School of Population and Global Health, University of Melbourne, Melbourne, Australia; e Burnet Institute, Melbourne, Australia; f Berman Institute of Bioethics, Johns Hopkins University, Baltimore, MD, USA; g STAR Fellows Department, Public Health Institute, Washington, DC, USA

**Keywords:** National immunization technical advisory group, NITAG, SAGE, COVID-19, Policy recommendations, Evidence-based decision-making

## Abstract

National Immunization Technical Advisory Committees (NITAGs) are tasked with the responsibility of guiding ministries of health and national immunization programmes in their policy development processes. Many NITAGs rely on evidence reviewed by the World Health Organization’s (WHO) Strategic Group of Experts (SAGE) on immunization and aim to adapt WHO’s recommendations to their respective contexts. This relationship took on exceptional importance since the onset of the COVID-19 pandemic, during which NITAGs have expressed a notable struggle to craft appropriate policies on population prioritization and vaccine utilization in the face of supply constraints and complex programmatic and delivery logistics.

This online survey was conducted to assess the usefulness of the SAGE guidance documents for COVID-19 vaccine policies and to examine the persisting needs and challenges facing NITAGs. Results confirmed that SAGE recommendations concerning COVID-19 vaccines are easy to access, understand, and adapt. They have been found to be comprehensive and timely under the data and time constrained circumstances confronting SAGE. The Global NITAG Network (GNN) appears to be the most popular vehicle for addressing questions among high income countries, in contrast to lower income countries who favour WHO Country or Regional Offices. NITAGs place much value on interaction with other NITAGs, which requires facilitation and could benefit from increased opportunities, especially within regions. It is further noted that some NITAGs have had to tackle issues during the pandemic not typically considered by SAGE, such as supply chain logistics and vaccine demand.

Learning from the COVID-19 experience offers opportunities to strengthen NITAGs and the pandemic recovery effort through the development of more concrete procedures and consideration of more varied types of data, including implementation effectiveness and uptake data. There is also an opportunity for an increasing involvement of Country Office WHO personnel to support NITAGs, while ensuring information and evidence needs of countries are adequately reflected in SAGE deliberations.

## Highlights

• NITAGs can rely on accessible and adaptable COVID-19 vaccine recommendations from WHO’s SAGE.
• NITAGs find interaction with fellow advisory groups within and beyond their regions beneficial.
• WHO country and regional offices can improve communications with NITAGs.
• The COVID-19 pandemic experience highlighted challenges in evidence-based policy development.
• NITAGs have opportunities to better address future pandemics and current recovery efforts.

## 1 Introduction

The World Health Organization (WHO) relies on the scientific reviews and expertise of its Strategic Group of Experts (SAGE) on immunization to advise and craft relevant evidence-based recommendations on policies and strategies, targeting all levels, from global down to sub-national decision-makers. Likewise, at country level, National Immunization Technical Advisory Committees (NITAGs) are tasked with the substantial responsibility of guiding ministries of health and national immunization programmes in their policy development processes. Many NITAGs rely on evidence reviewed by SAGE and aim to adapt WHO’s recommendations to their respective contexts.

During the course of 2021, the SAGE was convened eleven times through virtual meetings to discuss COVID-19 vaccination. For each of the vaccine products listed by the World Health Organization (WHO) for Emergency Use, interim recommendations and accompanying evidence to recommendation frameworks were issued. Additional SAGE outputs related to the COVID-19 Pandemic have been a *WHO-SAGE values framework for the allocation and prioritization of COVID-19 vaccination –* published in September 2020 – and the *WHO SAGE Roadmap for prioritizing uses of COVID-19 vaccines*- first issued in October 2020 and last updated in January 2022. SAGE has also published interim recommendations on COVID-19 vaccination of immunocompromised persons, booster vaccination, and heterologous schedules, in addition to issuing multiple interim technical statements to clarify varied policy issues [1].

NITAGs are both a technical resource and a deliberative body to advise national authorities and policy makers seeking to make evidence-based decisions [2]. NITAGs are considered an essential component of a functioning immunization system [3]. They are recommended to operate as science-focused bodies independent of both government policy makers and of external public health and development partners; the latter groups’ resource allocation functions are often captured in Inter-agency Coordination Committees. The primary purpose of NITAGs is to assist Ministries of Health through the provision of information and guidance required to develop appropriate policies that consistently reflect the latest evidence, address national public health needs and contextual realities, and help build public confidence. In recognition of these important benefits, the WHO has made the establishment and strengthening of NITAGs a priority [4], [17]. The 2018 World Health Assembly also reaffirmed the importance of NITAGs for country ownership and credibility of national immunization programmes. Consequently, the number of NITAGs has tripled since 2010, bringing the total in 2021 to 170 [5]. Of these, over 72% are considered fully functional^1^ [6], serving over 85% of the world’s population [7]. Nevertheless, The Global Vaccine Action Plan^2^ 2020 target of every country having established or being able to access a NITAG was not met [17], and the COVID-19 pandemic has highlighted new challenges and needs underpinning the important role of NITAGs [8].

During a Global NITAG Network (GNN) webinar on country-level COVID-19 policy successes and needs held in in June 2021, discussion confirmed that the most difficult task for NITAGs at that time, irrespective of the vaccine product under consideration or the national income level, was crafting appropriate policies on population prioritization in the face of supply constraints and complex programmatic and vaccine delivery logistics. Another significant challenge was the task of delivering multiple products with varying handling requirements and use recommendations, affecting both NITAGs charged with developing coherent policy and national immunization programmes confronted with their implementation.

In response to a call by SAGE for more clarity on the usefulness and applicability of SAGE COVID-19 related recommendations and to inform the deliberations of the COVID-19 Vaccines Working Group, a survey of NITAGs was developed. The overall objectives of this survey were to: (a) rapidly assess the usefulness of the SAGE products for COVID-19 vaccine policies and identify measures to enhance their uptake among NITAGs, (b) identify guidance and policy challenges facing NITAGs and National Immunization programs, including unmet needs in terms of evidence, guidance, and capacity, and (c) examine how these challenges compare across countries by regional and economic categories.

## 2 Methods

### 2.1 Survey design

The survey was carried out by the SAGE secretariat which, in addition to its role of leading all SAGE related activities, also has a wider coordination function concerning all matters of relevance to immunization policy at global, regional and country level. Each NITAG for whom WHO could access contact details was invited to select a single representative to complete a voluntary 4-part survey consisting of 29 questions. An initial section collected basic information about the NITAG and the country context. A second section was dedicated to accessibility, suitability and usability of COVID-19 vaccine policy outputs published by WHO. The third section aimed to collect insights on the role and process each NITAG has played or followed to develop COVID-19 vaccine policies. Lastly, there was a brief fourth section focusing on COVID-19 policy challenges that was intended to help rapidly identify stress points around issues of concern in vaccine policy development, as well as where vaccine policy gaps persist. The survey was available in English, French, Spanish and Russian (Annex 1).

In order to verify the survey was fit for purpose and to ensure all questions could be adequately interpreted and answered by NITAGs from broad-ranging geographic and economic settings, as well as levels of committee maturity, a brief piloting and validation of the survey was implemented, including the translated versions in Spanish and French. The survey was piloted among the six members of the GNN steering committee, representing each of the WHO regions^3^. The survey was assessed not to require full WHO ethics review by the WHO Research Ethics Review Committee as no personal patient data was collected given the system quality improvement objectives of the survey (ref.CERC.0144).

### 2.2 Data collection

A link to an online survey, created for maximum simplicity and efficiency through Microsoft Forms, was sent out in late November 2021 to a total of 98 NITAGs by email to both the secretariat lead and the chair of each advisory group, requesting a coordinated response and offering the option to complete a version in one of the four languages. The 98 NITAGs contacted included the sub-regional Immunization Technical Advisory Group for the Caribbean region (CITAG) that encompasses 22 countries and territories/13 WHO Member States [9], and the 74 registered GNN members, as well as additional NITAGs for whom contact information was available as a result of registration for SAGE meetings or communications with WHO regional office focal points. Regional office focal points were also encouraged to disseminate the survey to additional countries and to follow up with country offices to increase the response rate. NITAGs were given three weeks to respond to the survey which was estimated to require approximately 20 min to complete. Multiple reminders were sent out as the survey deadline approached. Only one response per NITAG was accepted as well as the collective response from the CITAG on behalf of Caribbean countries- operating as a single NITAG for the purposes of the survey. Additional data on regional categories, World Bank income-level, and NITAG functionality status (according to WHO criteria) were added to the dataset to allow for additional stratifications and comparisons, but these were not factors in the sample selection. Countries without a NITAG were not approached as they were determined to be beyond the scope of this survey.

### 2.3 Analysis

Full text responses in languages other than English were translated into English.

Descriptive statistical analysis was conducted using Microsoft Excel, and grouped thematically by survey section, as well as through regional and economic stratification. Qualitative findings were subjected to simple thematic analysis, based on the survey structure, with similar themes grouped together.

Given the small sample size and the self-selection permitted within the sampling methodology, inferential analysis was deemed inappropriate. While stratification of survey responses was systematically conducted per income classification and WHO region, only the most relevant results are commented on.

The results of the survey were presented to the SAGE Working Group for COVID-19 and to SAGE members, who expressed their satisfaction with the findings.

## 3 Results

### 3.1 Survey respondent profiles

A total of 44 responses (45% response rate) were included in the final analysis including nine in French and six in Spanish. No responses were received in Russian. The CITAG provided a single survey response on behalf of 13 WHO Member States (see Annex 2). As these countries include a variety of income levels, this response was not analyzed in any of the economic analysis, therefore appearing as the “NA” value in many of the figures below.

**Fig. 1:**
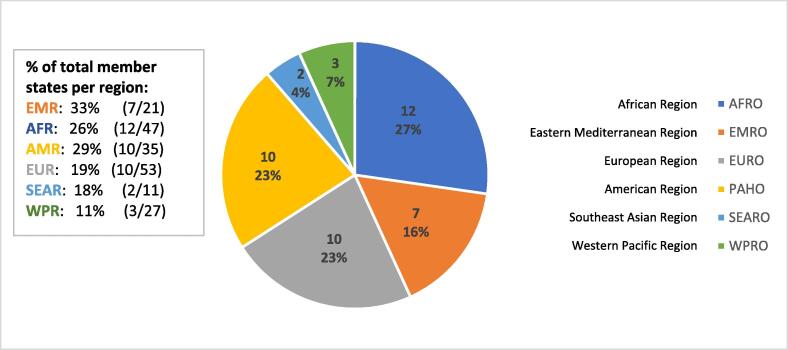
Survey participation across WHO regions.

**Fig. 2:**
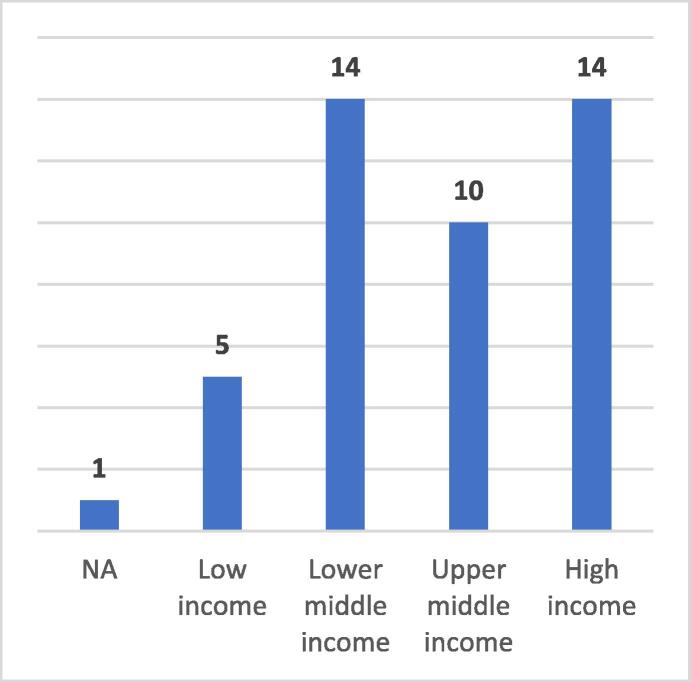
Distribution of responses per World Bank income classification.

**Fig. 3:**
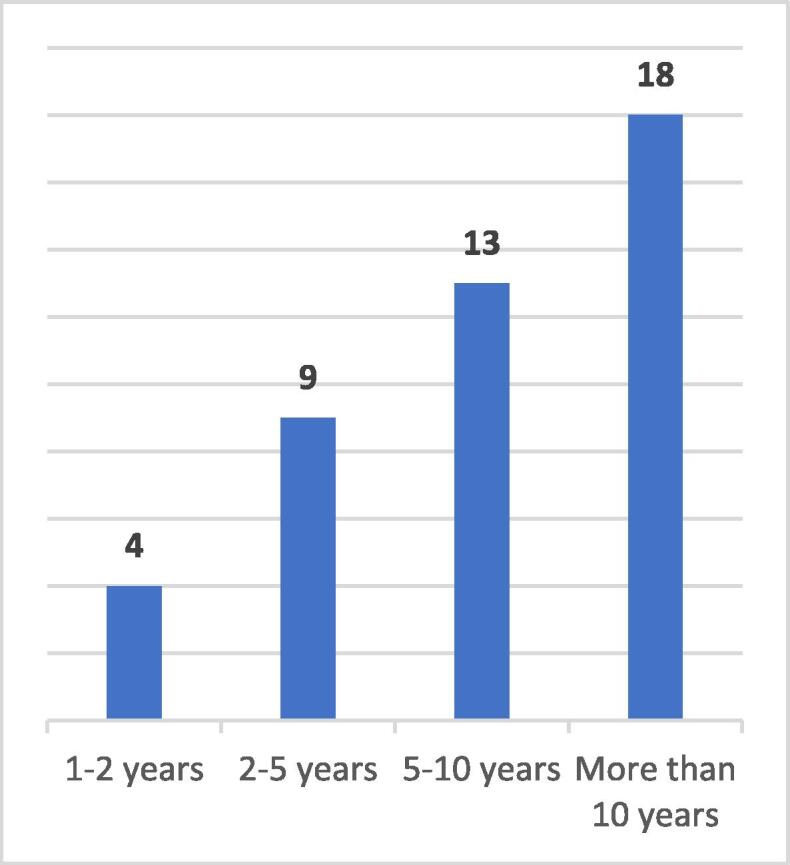
Distribution of responses per years NITAG has been active.

There was participation from across the six regions of WHO, as illustrated in Fig. 1, though proportionally, when taking into account the proportion with respect to the total number of Member States within a given region- as listed in the sub-table accompanying the pie chart in Fig. 1, there was more representation from the Eastern Mediterranean Region (EMR), the African Region (AFR), and the Region of the Americas (AMR). This was similarly the case in terms of population sizes represented per region, with 53% of the AMR population being covered by responding NITAGs, 44% of the AFR population, and 15% of the EMR population. For the European Region (EUR), this figure was 14%. The Southeast Asia (SEAR) and the Western Pacific (WPR) regions were the least represented, with only 4% and 2% of the respective regional populations being reflected in the responding NITAGs [10]. There was somewhat stronger representation among higher income countries, as well as long-standing NITAGs (Fig. 2, Fig. 3). Notably, 41% of responding NITAGs had been active for over ten years, all of which came from middle- or high-income countries. The responding NITAGs representing high income countries consisted of 32% of the study population. Only 11% of respondents were from low-income countries. Furthermore, 75% of the participating NITAGs qualified as fully functional by WHO definitions, with the longer the NITAG’s existence, the greater the likelihood of being fully functional. In total, approximately 18% of the world’s population was covered by the NITAGs that participated.

Of survey respondents, 42% met less than five times during 2021. A further 29% met between 5 and 12 times. And the remainder met on average at least once per month, with a subset of 5 NITAGs meeting at a frequency of more than twice per month. The number of years a NITAG was active did not appear to affect the frequency of meeting, nor did functionality. Participating NITAGs from upper middle- and high-income countries had a higher frequency of meetings (mean = 20) than those from lower middle- and low-income countries (mean = 8).

### 3.2 Accessing and using SAGE COVID-19 outputs

The survey results demonstrate a high level of participation by NITAGs in the virtual SAGE meetings that had been held throughout 2021. Eighty six percent of responding NITAGS reported attending at least one of the virtual SAGE meetings. The WHO website, which benefited from a structural update, was the most common method of accessing WHO/SAGE recommendations, followed by the GNN updates and emails from the SAGE Secretariat. All respondents found navigating the WHO website to access SAGE recommendations relatively easy. The majority of those NITAGs also considered SAGE interim recommendations easy to understand (93%) regardless of the country income level or longevity of the NITAG.

Most respondents found WHO/SAGE recommendations easy to adapt to their own country (64%), timely (75%), and with the related background documentation being sufficiently comprehensive (77%). However, respondents from the African and American regions expressed a greater preference for additional accompanying guidance from Regional Immunization Technical Advisory Groups (RITAGs) than those from other regions.

NITAGs from the Eastern Mediterranean and African Regions attributed a more important role to WHO Country Offices when it came to obtaining clarifications on global policy recommendations. In the European Region, the Region of the Americas, and the Western Pacific Region, there appeared to be a preference to rely on dialogue with the GNN and other NITAGs. Likewise, the GNN was the most popular mechanism for addressing questions and clarification among high income countries, in contrast to the lower income countries who favoured the WHO country office or WHO regional or sub-regional offices. A similar pattern was noted when comparing NITAGs with more than 10 years of existence- whose preference leaned towards the GNN- to those with only 1 to 2 years of existence- whose preference was mainly to turn to the WHO Country Office.

The survey also provided the opportunity for respondents to make suggestions on how to improve suitability and usability of WHO/SAGE Interim recommendations on COVID-19 vaccination. Among the 29 responses to this open-ended question, 38% suggested ways to improve how and where recommendations are communicated and disseminated – such as suggesting that more informative webinars be conducted and that recommendations be shared directly with Immunization programme staff and translated in a timelier manner. An additional 20% requested that recommendations take more into account the geographic and economic differences of Member States, but without going into more detail.

### 3.3 National COVID-19 vaccine policy development process

For 28 of respondents (64%), the NITAG was considered the main body advising on COVID-19 vaccine recommendations for the country. Thirty NITAGs (68%) indicated that the country had a dedicated COVID-19 vaccination committee in addition to the NITAG or as a replacement of the NITAG, though the question failed to allow for clarity on whether this qualified as a sub-committee or a separate working group, as well as on the particular objectives of said committee. Of these, only one third (10/45) had a formal mechanism of interaction between that group and the NITAG, even though all but three of these dedicated groups include at least one NITAG member.

In only eight (18%) responding countries did NITAG secretariat staffing capacity increase to accommodate the extra policy workload, even though in at least 82% of cases, multiple guidance statements had been issued on COVID-19 vaccination. Of those eight countries where staffing was increased to better address COVID-19 policy work, half were high-income countries, one was an upper middle-income country, and the remaining three were lower middle-income. None of the low-income countries reporting making staffing adjustments.

Among 25 (58%) responding countries from the full sample, the Ministry of Health consistently adopted NITAG recommendations. When this was not done, the main reasons cited was either programme or supply constraints. Only three responders (7%) stated their Ministry of Health never followed NITAG recommendations, though in all three cases, the NITAG was not the main advisory body for COVID-19 vaccine policies. Among the 28 NITAGs responding that they were the main advisory body for COVID-19 vaccine policies, 75% saw their Ministry of Health consistently following their recommendations. Neither the region nor the income level appeared to be a factor.

Countries who relied on the NITAG as their main advisory body for COVID-19 vaccine policy development tended to have a fully functional NITAG, per the process indicators listed above.

WHO/SAGE Interim Recommendations were the most popular source of information among responding NITAGs to inform policy development. Additionally, 86% reported relying on the Evidence to Recommendation (EtR) approach and close to 40% have developed their own Grading of Recommendations, Assessment, Development and Evaluation (GRADE) methods and evidence tables. The given country’s economic level did not appear to be a factor in these activities. However, 64% of participating NITAGs also consider what other NITAGs in their region were recommending – this was especially the case for NITAGs in the European Region and in Region of the Americas. The majority of the responding countries within the Americas and Europe also appear to consider recommendations outside of their respective region.

**Fig. 4:**
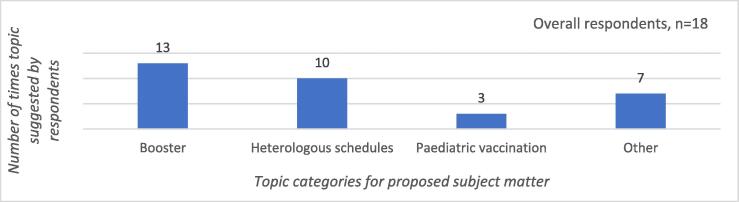
Key policy guidance missing from SAGE in Nov/Dec 2021.

Eighteen (41%) respondents suggested that policy guidance from SAGE was lacking for certain topics at the time of the survey. As Fig. 4 illustrates, among those topics, boosters were mentioned most, followed by heterologous schedules^4^.

**Fig. 5:**
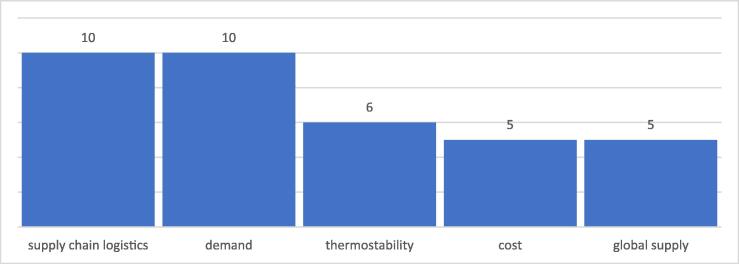
Top Five examples of NITAG policy concerns not covered by SAGE recommendations.

Just under half (46%) of the participating NITAGs indicated having additional policy concerns that SAGE doesn’t typically consider. The top examples are listed in Fig. 5, with supply chain logistics and vaccine service demand being cited most frequently. The distribution of NITAGs expressing an interest in additional policy issues was seen across the whole sample and did not suggest a more pronounced need in a geography or income group.

### 3.4 COVID-19 vaccine policy challenges

**Fig. 6:**
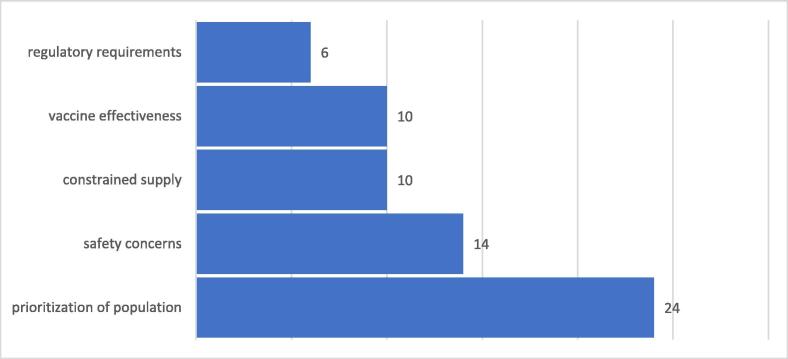
Top Five cited issues considered during COVID-19 vaccine decision-making.

When respondents were asked to select three issues that were most important in developing COVID-19 vaccine recommendations, the prioritization of populations emerged as the most common, followed by safety concerns (Fig. 6). An open question about key policy enablers suggested that interaction with other NITAGs and improved access to WHO global-level recommendations and evidence were the most significant aids among participating countries. Lastly, while evidence was the greatest driver of policy (87%), over half of the responding NITAGs (55%) acknowledged that political pressures played a role in the policy development process.

## 4 Discussion

### 4.1 Survey respondent profiles

Considering the length of the survey and the significant workload NITAGs across the world have been facing due to the pandemic, the 45% response rate was assessed as highly satisfactory [11]. The survey was however prone to some selection bias. As noted, high-income countries are slightly more represented than middle- and low-income countries, possibly due to more human resources being available to respond to external requests. However, taking into account global percentages, the sample was nevertheless considered sufficiently representative across the income groups (cf. Annex 3).

Similarly, longer-standing NITAGs have had more opportunity to organize themselves and effectively mobilize a more engaged approach to interaction with WHO initiatives such as elective surveys. However, this also speaks to a notable need for proactive efforts by WHO to support and engage the lesser-resourced or more recently established NITAGs, as it is these very NITAGs who appeared most in need and most interested in the kind of guidance that is offered by SAGE and the GNN.

### 4.2 SAGE COVID-19 vaccines outputs

The survey results suggest that the SAGE secretariat was effective in helping NITAGs have equitable access to SAGE policy discussions and outputs concerning COVID-19 vaccines. There were multiple complementary efforts that ensured that regardless of the dissemination approach, NITAGs easily accessed and understood WHO/SAGE recommendations and other policy documents for the most part, irrespective of their longevity or their choice of whether to approach COVID-19 policy development directly or by way of a separate dedicated committee. The comments on timeliness (boosters and heterologous schedules) emphasized the need for SAGE to be as rapid as possible in providing advice.

WHO SAGE recommendations were seen as adaptable, and all respondents could identify a reliable source of help in the process of interpreting and applying them to their own context.

The survey results nevertheless suggest there might be room for contextualizing further global recommendations at a regional and country level, as shown from the interest expressed by respondents for WHO Country Office to provide clarifications on globally-issued policies, and to improve adaptation and dissemination, considering differences in resources and need that may warrant special programs such as outreach strategies. Regional WHO offices supported by their respective RITAG and WHO country offices are well placed to address those needs which appeared to be most pronounced among the low- and lower-middle income countries.

In this context, offering more opportunities for regional-level exchanges could be of benefit, as well as strengthening routine two-way communication channels between NITAGs and WHO regional offices, in liaison with SAGE and GNN, following the European model discussed by Mosina et al [12]. Even though NITAGs for the most part found SAGE outputs to be timely, it is recognized that both NITAGs and SAGE have had to balance the need to make recommendations before the science on a particular topic was fully available and respond urgently to the pandemic.

### 4.3 National COVID-19 vaccine policy development process

A NITAG is only as effective as the corresponding health authorities’ ability and willingness to adopt its recommendations. In the context of the COVID-19 pandemic, many expectations were placed on NITAGs and for the most part, those who participated in this survey were able to rise to the challenge. The results of this survey suggest that NITAGs were largely trusted by Ministries of Health as a credible and reliable source of guidance on COVID-19 vaccine decision-making and can therefore be considered a good vaccine advisory model for future pandemics or other vaccine related policy questions.

As future pandemic preparedness gains momentum and feedback for continuous quality improvement already gaining traction, the roles of NITAGs in support of evidence-based vaccine policy development and decision-making merit further exploration. Learning from the COVID-19 experience offers opportunities to strengthen NITAGs through the development of more concrete standard operating procedures. One such example was the call for guidance on heterologous schedules. In the absence of robust data, WHO was initially forced to hold off on specific recommendations. However, as programme needs and realities outpaced the clinical research on this subject, in tandem with a volatile national media context and changing national priorities, SAGE addressed this policy issue through the publication of subject-specific interim guidance [1]. The latter relied on a risk–benefit assessment and directly took into consideration programmatic concerns such as the lack of availability of the same vaccine product in settings with limited or unpredictable supply. Similarly, the issue of vaccine thermostability affecting the shelf life of vaccines and the ability of programmes to use stock while still honouring prioritization guidance points to the critical role implementation data can play in policy development and revisions, as well as how vaccine uptake must factor into policy deliberations.

### 4.4 COVID-19 vaccine policy challenges

There was clear indication that NITAGs appreciated the support and guidance provided by the SAGE Secretariat. In addition, a high value seemed to be placed on interaction with other national advisory groups, be it within their same region or beyond, as this can shed light on how other countries have handled challenges or provide access to data from similar populations. This underscores a valued role for the GNN that could be leveraged further to coordinate more effectively across regions and to seek greater engagement from regional focal points with WHO regional offices as well as within RITAGs.

While many higher income countries do not have WHO country offices, particularly in Europe (where 45% of Member States do not have a WHO office) and the Americas (23% of Member States do not have a WHO office) [13], survey results nevertheless suggest a potentially stronger role for WHO country offices for clarification of WHO/SAGE policies in less resourced countries, which can strengthen the value and uptake of NITAG recommendations by Ministries of Health. In addition, WHO country offices can ensure that programme challenges are taken into account in the decision-making of NITAGs. Lastly, country-level WHO personnel can serve as an important voice to ensure the information and evidence needs of countries are adequately reflected in SAGE deliberations.

In order to keep the survey short, it was not possible to include questions allowing for an assessment of NITAGs’ more specific COVID-related needs, nor any about competing demands, pandemic-related stress and pressure, or even the possible satisfaction and improved visibility felt by NITAGs working during a global pandemic. It is well documented that national immunization programmes have suffered important setbacks during this pandemic, but NITAG insights have not been documented [14]. Likewise, unpublished WHO reports have revealed that most NITAGs were entirely focused on COVID-19 recommendations for the better part of the pandemic to date, the only possible exception being influenza. Not having the needed resources to address other pathogens during the pandemic inevitably undermined the advisory group’s capacity to support overall immunization objectives of a national programme.

While the trajectory of the COVID-19 pandemic remains uncertain, national immunization programmes continue to be confronted with difficult decisions on appropriate COVID-19 vaccination policies, including determining optimal product selections for their specific population and context, as well as the best use of limited resources in the face of other vaccine program needs. A variety of guidance is available to facilitate such tasks, including the reference documents and tools available through the NITAG resource center [5] or the TechNet Library of immunization resources [16]. These tools could improve the reflection of programmatic challenges such as supply chain and prioritization difficulties in policy decisions, both being noted by NITAGs responding to this survey as areas of need.

## 5 Limitations

The survey only allowed one respondent per country which introduced the risk of reporter bias. As noted above, there was a higher representation of high-income country and longer-standing NITAGs, which may have biased responses to reflect the view of more functional groups. Nevertheless, on the basis of the distributions detailed in Annex 3, the authors considered the responses were sufficiently representative to enable the drawing of some conclusions, despite limiting broader insights on the specific needs and challenges of lower-income countries.

Limited survey pre-testing did not allow for questions to be fully tailored to fit very different contexts and experiences. Had there been further iteration of the questionnaire, this might have mitigated the varied interpretation of certain survey questions. The variation in how a few of the questions in section III were interpreted undermined some of the potential for analysis on sub-group optimization and impact. How NITAGs perceive and define a separate or dedicated vaccine sub-committee therefore remains unclear. It was also difficult to determine the more specific factors driving policy uptake by Ministries of Health. These could have informed an improved approach to how NITAGs are supported, and how their recommendations are made.

Survey data was analyzed together with data obtained from the annual WHO-UNICEF Joint Reporting Form (JRF) in the first half of 2022. The JRF is usually submitted by countries to WHO and UNICEF between January and April for the previous year. Data for 2021 was therefore not available until June 2022 preventing analysis on the impact of the COVID-19 Pandemic on overall NITAG functionality, as defined by the six criteria that are reported on as performance indicators through the JRF [7].

## 6 Future research

Regular dialogue and assessment of NITAG processes, needs, and challenges are central mechanisms to ensuring NITAGs are functional, impactful and remain fit for purpose. Future studies should be strengthened by complimentary focus groups or qualitative interviews that would allow for more detailed collection of qualitative evidence and the validation of these preliminary survey results. Subsequent periodic surveys could aim for further delineation of NITAG stressors, needs from WHO and SAGE in different contexts and geographic settings, and how to optimize efficiency in their work. Lastly, in view of the limited representation from low-income countries in this survey, additional efforts are required to assess policy and decision-making challenges in the face of constrained resources.

The specific drivers of the uptake of policy recommendations must be studied more closely. The outcomes from this survey indicate that programme and supply constraints have been important considerations shaping recommendations. It could therefore be a good lesson to take forward that evidence and data on such subjects be made available to NITAGs and MOHs whenever possible, to strengthen the decision-making process. For example, evidence from implementation research shedding light on supply logistics and demand could prove beneficial.

As COVID-19 situation continues to occupy a significant place in NITAG and Immunization programme agendas and future emergencies and/or pandemics may leverage this infrastructure, it would be worth examining further to what extent NITAGs have been strengthened, provided further resources to improve their function and solicited over time for continued guidance on non-COVID-19 vaccine matters. Further data on how these investments and ongoing dialogue have impacted the structure and function of NITAGs in their performance and capacity.

## 7 Conclusions

Vaccination took center stage within public discourse in 2021 like in no other year in history. With pressure mounting to curb the COVID-19 pandemic and bring both the spread of SARS-CoV-2 and emerging variants under control in order to reduce the rate of hospitalization and deaths, countries were scrambling to secure vaccines and implement rapid roll-outs. Consequently, national immunization programmes frequently found themselves handling up to four or more COVID-19 vaccines from multiple technology platforms, each with their own level of efficacy, particular set of safety concerns, and evidence gaps, as well as varying storage and handling requirements. In addition to coping with erratic and unpredictable supply, countries were also confronted with the complexity of an epidemic with a rapidly mutating virus, the pressure to take decisions based on incomplete or patchy evidence, the challenges around COVID-19 vaccine acceptance and demand and communication, a limited and over-stretched health workforce, an ill-equipped infrastructure particularly for wide spread vaccine delivery, and difficulties in identifying, prioritizing, and adequately mobilizing or accessing targeted adult populations.

The COVID-19 pandemic has placed unparalleled pressure on NITAGs to support country policy and programme decisions, making them far more visible in society than they previously had been and the object of both important expectations and extraordinary scrutiny and pressure. Ensuring NITAGs are receiving the technical guidance and evidence insights they need is therefore more critical than ever. There are potential benefits to be gained through increased regional engagement and support in policy development at country level. Many NITAGs, especially in low- and middle-income countries, would gain from strengthened collaboration and exchange with other NITAGs within their regions and beyond, including across their language networks, for the purposes of obtaining insights which can inform policy development. The critical role that WHO can play, particularly at Country Office level, must also be acknowledged and further leveraged, such as for dissemination of WHO guidance and sharing programmatic data that could reinforce policy decisions. These lessons and further strengthening activities will help countries in the face of future outbreaks and pandemics, as well as for the sustainment and recovery of routine programmes negatively affected by events of significant disruption potential.

## Declaration of Competing Interest

The authors declare that they have no known competing financial interests or personal relationships that could have appeared to influence the work reported in this paper.

## Data Availability

The data that has been used is confidential.

## Footnotes

1 According to the six process criteria collected annually by WHO [15]

2 Superseded by the Immunization Agenda 2030 (https://www.immunizationagenda2030.org/, accessed 21 September 2022)

3 https://www.nitag-resource.org/network/steering-committee

4 The use of heterologous schedules is commonly referred to as a “mix and match approach”, which was the term used as a suggestion in the relevant survey question.
