# Supplementary material for: COVID-19 vaccine policy development in a sample of 44 countries – Key findings from a December 2021 survey of National Immunization Technical Advisory Groups (NITAGs)
Source: Vaccine. 2023 Jan 16;41(3):676–83. doi: 10.1016/j.vaccine.2022.11.029 (PMC9671626; doi:10.1016/j.vaccine.2022.11.029)
Supplement: Supplementary data 1 [file mmc1.docx]

**ANNEX 1: SURVEY QUESTIONNAIRE**

* Obligatory questions

**Basic information on the NITAG**

## Please indicate the country your NITAG represents *

## Has your NITAG been active for: *

## 1-2 years

## 2-5 years

## 5-10 years

## More than 10 years

1. In 2021, how many meetings (virtual or in-person) has the NITAG held where COVID-19 vaccination has explicitly been part of an agenda point? *

# SAGE COVID-19 VACCINES OUTPUTS

# *Objective*: Inform SAGE guidance development process to ensure documents are as useful as possible to NITAGS and easily accessible.

## Since the pandemic, has at least one NITAG representative of your country attended any virtual SAGE meetings? *

- Yes
- No
- I don’t know

## If no, what is/are the reason(s)?

- No time
- Time zone issue
- No translation in your native language
- Not aware NITAG representative can participate
- No exchange possible with SAGE members
- Other

## How do you access the WHO/SAGE recommendations and other policy documentation? *

- by checking the WHO website
- emails from the SAGE secretariat
- emails from regional office/IST
- the GNN email updates
- Google search
- Other

## When searching for a specific policy recommendation, how easy or difficult is it for you to find it on the WHO/SAGE website? * (on a scale of 1/difficult to 10/easy)

## How strongly do you agree or disagree with the following statements? (select from: strongly disagree; Disagree; Neutral; Agree; Strongly agree) *

## I find the WHO/SAGE product specific recommendations easy to understand

## I find the WHO/SAGE product specific recommendations timely

## I find the WHO/SAGE and background document of product specific recommendations comprehensive

## I find WHO/SAGE recommendations easily adaptable to my country context

## I find WHO/SAGE recommendations more helpful when they come with advice from my RITAG

## When you have questions or need clarification to better understand the WHO/SAGE policies, where do you seek this information from? tick all that apply *

## WHO country office

## WHO Regional Office/ISTs

## WHO headquarters

## Global NITAG Network (GNN)

## Other NITAGs

## Manufacturers

## Others

1. What could be done to enhance the usefulness of WHO/SAGE interim recommendations?

# NATIONAL COVID-19 VACCINE POLICY DEVELOPMENT PROCESS

*Objective*: Understand role and process each NITAG has played/followed to develop COVID-19 vaccine policies

1. Was the NITAG the main advisory body to advise on COVID-19 vaccine policies in your country? *

## In addition to the NITAG or as a replacement of the NITAG, was there a dedicated COVID-19 vaccination committee (with a clear mandate and defined terms of reference) for the MoH?

- Yes
- No

## If yes, were any NITAG members also members of this committee?

- Yes
- No
- I don’t know

## If yes, is there a formal interaction mechanism between the NITAG and the COVID-19 committee (e.g. regular meetings, common reporting lines…)?

- Yes
- No
- I don’t know

## Have staffing capacities for the secretariat of your NITAG increased to face the COVID-19 vaccines policy workload? *

- Yes
- No
- I don’t know

## In 2021, how many guidance statements has your NITAG issued on covid- 19 vaccination? *

- None
- One
- At least 2
- 5+
- More than 10

## Has the MoH followed the NITAG recommendations:

- Consistently
- Sometimes
- Never

## If sometime or never, please elaborate:

1. When developing COVID-19 vaccine policies, which of the following information do you use (check all that apply): *

- the WHO/SAGE interim recommendations
- the WHO/SAGE annexes (GRADE and evidence-to-recommendation (EtR) tables) to the recommendations
- the WHO/SAGE background documentation
- the RITAG recommendations
- None of the above

## When developing COVID-19 vaccine policy recommendations, does your NITAG: * (Select between hardly ever and most of the time for each of the following statements)

- Use the Evidence to Recommendation approach to make your own policy recommendations?
- Develop your own GRADE tables?
- Consider other NITAGs’ recommendations from your region?
- Consider other NITAGs’ recommendations from other regions?

## When your NITAG was issuing or revising recommendations, how often (on frequency scale of 1/never to 10/always) were SAGE interim recommendations available?

## When your NITAG was issuing or revising recommendations, how often (on frequency scale of 1/never to 10/always) was regional guidance available?

## When your NITAG was issuing or revising recommendations, how often (on frequency scale of 1/never to 10/always) was regional guidance available? *

## If policy guidance from SAGE is still missing, on which specific topic(s): e.g. target population/product/timing second dose/ mix and match/ booster…

## Are there additional policy concerns and types of evidence that your NITAG takes into account that are not typically covered by SAGE?

- Yes
- No

## If yes, please select the most common examples

- global supply
- supply chain logistics
- thermostability
- ease of delivery
- vaccine storage
- packaging and presentation
- cost
- demand
- other

# COVID-19 VACCINE POLICY CHALLENGES *Objective*: Determine main tension points in terms of issues of concern and challenges in vaccine policy development, as well as where vaccine policy gaps persist

## In your country, please select the top 3 issues you had to take into account when developing COVID-19 vaccine recommendations: *

- equity
- prioritization of population
- constrained supply
- regulatory requirements
- managing multiple vaccines
- vaccine hesitancy
- implementation issues
- political demands
- safety concerns
- vaccine effectiveness
- others

## What have been the enablers to formulate or update your countries COVID-19 vaccine policies? (such as availability of training resources, collaboration between national committees, increased accessibility to WHO/SAGE resources, etc.)

1. In your opinion, were COVID-19 vaccine policies in your country driven predominately by

- Evidence
- Politics
- Both evidence and politics
- What funders supplied
- Other

## Please feel free to use the space below in case of any other information or requests you would like to share with the SAGE secretariat

- end of survey -
