# Supplementary material for: COVID-19 vaccine policy development in a sample of 44 countries – Key findings from a December 2021 survey of National Immunization Technical Advisory Groups (NITAGs)
Source: Vaccine. 2023 Jan 16;41(3):676–83. doi: 10.1016/j.vaccine.2022.11.029 (PMC9671626; doi:10.1016/j.vaccine.2022.11.029)
Supplement: Supplementary data 2 [file mmc2.docx]

**ANNEX 2: List of countries represented by the responding NITAGs**

ALBANIA

ANGOLA

ARGENTINA

ARMENIA

AUSTRALIA

BELGIUM

BRUNEI DARUSSALAM

BURKINA FASO

CAMEROON

CANADA

CHILE

COLOMBIA

DJIBOUTI

ECUADOR

FINLAND

FRANCE

GEORGIA

HAITI

HONDURAS

LATVIA

LEBANON

MALAWI

MONGOLIA

MOROCCO

MYANMAR

NAMIBIA

NIGER

NIGERIA

NORWAY

PALESTINE

PERU

RWANDA

SENEGAL

SOUTH AFRICA

SRI LANKA

SUDAN

SWEDEN

THE NETHERLANDS

TUNISIA

UGANDA

UNITED ARAB EMIRATES

UNITED STATES

ZIMBABWE

Caribbean Immunization Technical Advisory Group (**CITAG**) : Member states

ANTIGUA AND BARBUDA

BAHAMAS

BARBADOS

BELIZE

DOMINICA

GRANADA

GUYANA

HAITI

JAMAICA

SAINT KITTS AND NEVIS

SAINT LUCIA

SAINT VINCENT AND THE GRENADINES

SURINAME

TRINIDAD AND TOBAGO
