# Supplementary material for: COVID-19 vaccine policy development in a sample of 44 countries – Key findings from a December 2021 survey of National Immunization Technical Advisory Groups (NITAGs)
Source: Vaccine. 2023 Jan 16;41(3):676–83. doi: 10.1016/j.vaccine.2022.11.029 (PMC9671626; doi:10.1016/j.vaccine.2022.11.029)
Supplement: Supplementary data 3 [file mmc3.docx]

**ANNEXE 3: Detail of survey response status by WHO region and income level.**

|  | **No. (%) of WHO Member States*** | **No. (%) of NITAGs who received the survey** | **No. (%) of NITAGs who replied to the survey** |
| --- | --- | --- | --- |
|  |  |  |  |
| **AFR** | **46 (100%)** | **29 (63%)** | **11 (37.9%)** |
| high income | 1 (2.2%) | 1 (3.4%) | 0 (0%) |
| upper middle | 6 (13.0%) | 3 (10.3%) | 1 (9.1%) |
| lower middle | 18 (39.1%) | 11 (37.9%) | 5 (45.5%) |
| low income | 21 (45.7%) | 14 (48.3%) | 5 (45.5%) |
|  |  |  |  |
| **AMR** (excluding “CITAG countries”)* | **22 (100%)** | **16 (72.7%)** | **9 (56.3%)** |
| high income | 5 (22.7%) | 5 (31.3%) | 4 (44.4%) |
| upper middle | 11 (50.0%) | 8 (50%) | 3 (33.3%) |
| lower middle | 5 (22.7%) | 3 (18.8%) | 2 (22.2%) |
| N/A | 1 (4.6%) | 0 (0%) | 0 (0%) |
|  |  |  |  |
| **EMR** | **22 (100%)** | **16 (72.7%)** | **7 (43.8%)** |
| high income | 6 (27.3%) | 3 (18.8%) | 1 (16.7%) |
| upper middle | 4 (18.2%) | 4 (25%) | 1 (16.7%) |
| lower middle | 7 (31.8%) | 7 (43.8%) | 3 (50%) |
| low income | 5 (22.7%) | 2 (12.5%) | 1 (16.7%) |
|  |  |  |  |
| **EUR** | **53 (100%)** | **23 (43.4%)** | **10 (43.5%)** |
| high income | 32 (60.4%) | 18 (72%) | 7 (58.3%) |
| upper middle | 17 (32.1%) | 5 (20%) | 3 (25%) |
| lower middle | 4 (7.5%) | 0 (0%) | 0 (0%) |
|  |  |  |  |
| **SEAR** | **11 (100%)** | **9 (81.8%)** | **3 (33.3%)** |
| upper middle | 2 (18.2%) | 2 (22.2%) | 0 (0%) |
| lower middle | 8 (72.7%) | 7 (77.8%) | 3 (100%) |
| low income | 1 (9.1%) | 0 (0%) | 0 (0%) |
|  |  |  |  |
| **WPR** | **27 (100%)** | **4 (14.8%)** | **3 (75%)** |
| high income | 9 (33.3%) | 2 (50%) | 2 (66.7%) |
| upper middle | 5 (18.5%) | 1 (25%) | 0 (0%) |
| lower middle | 11 (40.7%) | 1 (25%) | 1 (33.3%) |
| N/A | 2 (7.5%) | 0 (0%) | 0 (0%) |
|  |  |  |  |
| **Global** | **181 (100%)** | **97 (53.6%)** | **43 (44.3%)** |
| high income | 53 (29.3%) | 29 (29.9%) | 14 (32.6%) |
| upper middle | 45 (24.9%) | 23 (23.7%) | 8 (18.6%) |
| lower middle | 53 (29.3%) | 29 (29.9%) | 15 (34.9%) |
| low income | 27 (14.9%) | 16 (16.5%) | 6 (14%) |
| N/A | 3 (1.6%) | (0%) | (0%) |

* There are in additional 13 WHO Member States in the Caribbean (AMR) served by a common NITAG: the CITAG. The CITAG provided a single coordinated response to the survey, on behalf of all its affiliated member countries and territories.
